# *In Vivo* Foot and Ankle Kinematics During Activities Measured by Using a Dual Fluoroscopic Imaging System: A Narrative Review

**DOI:** 10.3389/fbioe.2021.693806

**Published:** 2021-07-19

**Authors:** Dongqiang Ye, Xiaole Sun, Cui Zhang, Shen Zhang, Xini Zhang, Shaobai Wang, Weijie Fu

**Affiliations:** ^1^ School of Kinesiology, Shanghai University of Sport, Shanghai, China; ^2^ Shandong Institute of Sport Science, Jinan, China; ^3^ Key Laboratory of Exercise and Health Sciences of Ministry of Education, Shanghai University of Sport, Shanghai, China

**Keywords:** dual fluoroscopic imaging system, *in vivo* kinematics, foot and ankle, ankle ligament sprain, functional flat foot

## Abstract

Foot and ankle joints are complicated anatomical structures that combine the tibiotalar and subtalar joints. They play an extremely important role in walking, running, jumping and other dynamic activities of the human body. The *in vivo* kinematic analysis of the foot and ankle helps deeply understand the movement characteristics of these structures, as well as identify abnormal joint movements and treat related diseases. However, the technical deficiencies of traditional medical imaging methods limit studies on *in vivo* foot and ankle biomechanics. During the last decade, the dual fluoroscopic imaging system (DFIS) has enabled the accurate and noninvasive measurements of the dynamic and static activities in the joints of the body. Thus, this method can be utilised to quantify the movement in the single bones of the foot and ankle and analyse different morphological joints and complex bone positions and movement patterns within these organs. Moreover, it has been widely used in the field of image diagnosis and clinical biomechanics evaluation. The integration of existing single DFIS studies has great methodological reference value for future research on the foot and ankle. Therefore, this review evaluated existing studies that applied DFIS to measure the *in vivo* kinematics of the foot and ankle during various activities in healthy and pathologic populations. The difference between DFIS and traditional biomechanical measurement methods was shown. The advantages and shortcomings of DFIS in practical application were further elucidated, and effective theoretical support and constructive research direction for future studies on the human foot and ankle were provided.

## Introduction

The human foot consists of 26 bones and 33 joints and is connected to the lower limbs through the ankle joint (Kessler et al., 2019). The foot and ankle are active within six degrees of freedom (6DOF) of translational and rotational motion and are important for standing, walking, running, jumping, climbing and other movements that are closely related to athletic performance, growth and development, ageing, fall risk and prevention and disease treatment. More than one million people in the United States suffer from impaired function every year because of musculoskeletal problems in the foot and ankle joints (e.g., flat feet, stress fractures, ankle instability, Achilles tendonitis and plantar fasciitis); these injuries result in approximately $1.2 billion of healthcare expenditures and nearly $10 billion of inconsequential loss (Belatti and Phisitkul, 2014; Adal et al., 2020). However, the mechanism of these acute or chronic injuries remain poorly addressed. Studying the characteristics of movement in the foot and ankle joints is of great importance to address the above injuries, promote an in-depth and accurate understanding of foot and ankle kinematics and provide a basis for solution proposals.

Traditional foot and ankle kinematic measurements, such as high-speed infrared motion capture systems, can calculate joint motion from the trajectory of reflective markers pasted onto the human body surface and are widely used in the kinematic studies of human segments (Caravaggi et al., 2011; De Mits et al., 2012). However, high-speed motion capture systems lack precision in the observation of real skeletal motion due to the influence of marker placement and related factors, such as skin, soft tissue vibration and movement artefacts (Fiorentino et al., 2016a; Fiorentino et al., 2016b). A previous study found an error of 2.7–14.9 mm between the position of the reflective marker and the bony landmarks of the foot and ankle when measuring the ankle joint in the neutral and rotational state (Maslen and Ackland, 1994). Alternatively, researchers have performed cadaveric studies or implanted steel beads in living bodies to calculate joint movements (Lundgren et al., 2008; Zhu and Li, 2012). Nevertheless, the dynamic conditions of cadavers without autonomous neural control and muscle activation are different from those of living bodies (List et al., 2012). Steel beads are highly invasive and susceptible to causing infection in living bodies, affect walking patterns and cause ethical problems (Bey et al., 2006; Giphart et al., 2012). Therefore, developing a new technology that can break through these precision limitations has become a goal of biomechanics and biomedical engineering.

A 2006 study was the first to adopt a dual fluoroscopic imaging system (DFIS) to quantify the movement rule of the internal structure of the foot and ankle accurately (de Asla et al., 2006). DFIS can capture the dynamic motion of joints *in vivo* without introducing error from the relative motion of soft tissues (Torry et al., 2011; Zhu et al., 2012). In contrast to traditional kinematic measurement technologies, DFIS is a noninvasive technology with high compatibility. Its measuring precision in the determination of joint position and capture of 6DOF motion in bony structures at different speeds is on the submillimetre level (Cross et al., 2017). In actual application, DFIS has been used to solve the problem of joint localisation (hip joint and symphysis pubis) during surgery (James et al., 2018). It has also been applied to evaluate rehabilitation indices, such as biomechanical changes after joint replacement and spinal fusion (Klemt et al., 2020; Zhou et al., 2021), and *in vivo* joint motion characteristics, such as the 6DOF motion of the knee and ankle (Cao et al., 2019c; Li et al., 2019). Given the above, this method can be utilised to quantify the movement in the single bones of the foot and ankle and analyse different morphological joints and complex bone positions and movement patterns within these organs. The application of DFIS in the field of medicine and biomechanics thus provides a new perspective for the noninvasive and accurate analysis of the *in vivo* kinematics of the foot and ankle.

Considering that DFIS is a new technology, the integration of existing single DFIS studies has great methodological reference value for future research on the foot and ankle. This narrative review aimed to evaluate existing studies that used DFIS to measure the *in vivo* kinematics of the foot and ankle during various activities in healthy and pathologic populations. The advantages and shortcomings of DFIS in practical application were further elucidated, and effective theoretical support and constructive research direction for future studies on the human foot and ankle were provided.

## Literature Search Methodology

**FIGURE 1:**
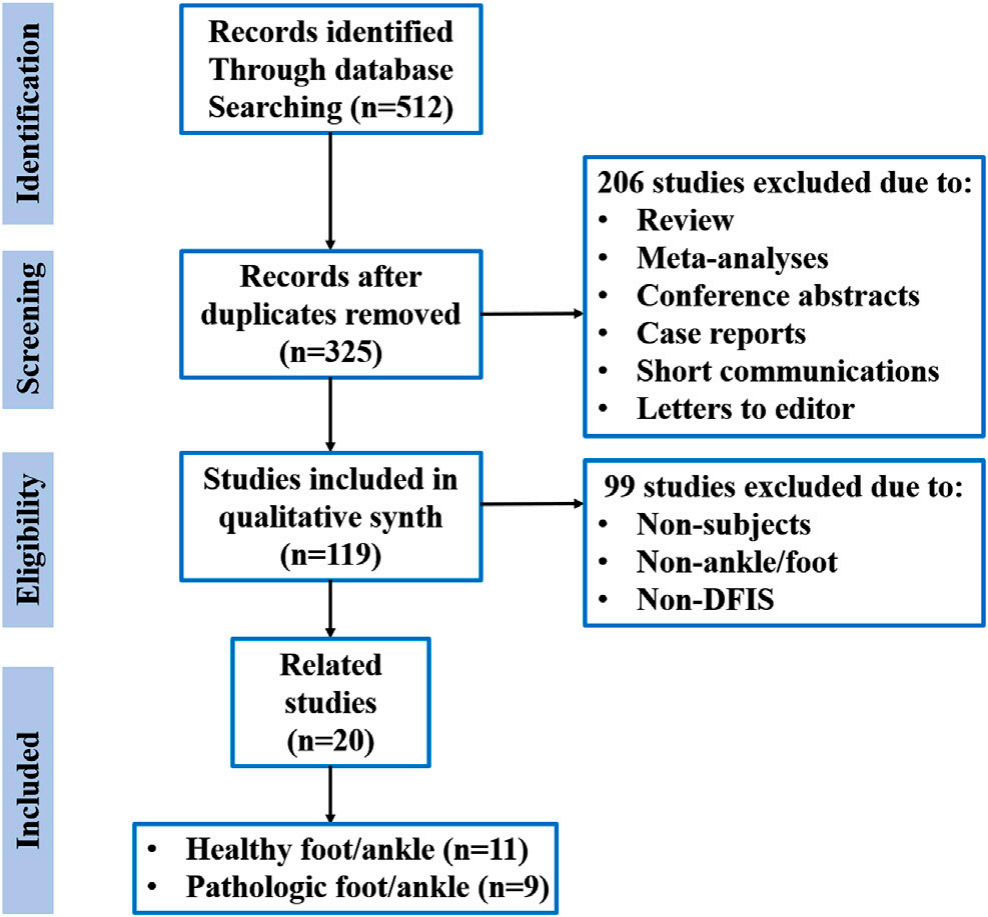
Literature search and study selection.

A standardised electronic literature search strategy was adopted *via* PubMed, Web of Science and EBSCO databases by using the keyword combinations “dual fluoroscopy,” “biplane fluoroscopy,” “biplanar video radiography,” “biplanar video fluoroscopy,” “biplanar fluoroscopy,” “biplane radiography,” “biplane X-ray system” or “biplane X-ray fluoroscopy” and “ankle” or “foot” and PUBYEAR from inception to January 2021. All articles were inputted into Endnote to eliminate duplicates. In the first analysis by abstract, reviews and meta-analyses, conference abstracts, case reports, short communications and letters to the editor were excluded. If the article met the criterion of the proposed review for DFIS, ankle joint, foot and participants, its full text was accessed and read in its entirety. Figure 1 summarises the search and selection processes.

## Dual Fluoroscopic Imaging System

### Development

**FIGURE 2:**
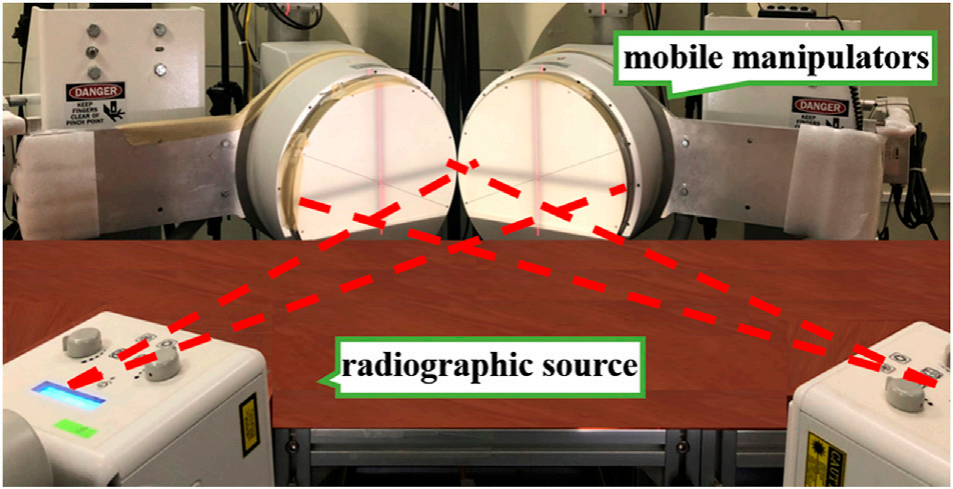
High-speed dual fluoroscopic imaging system (Shanghai University of Sport, Shanghai).

**FIGURE 3:**
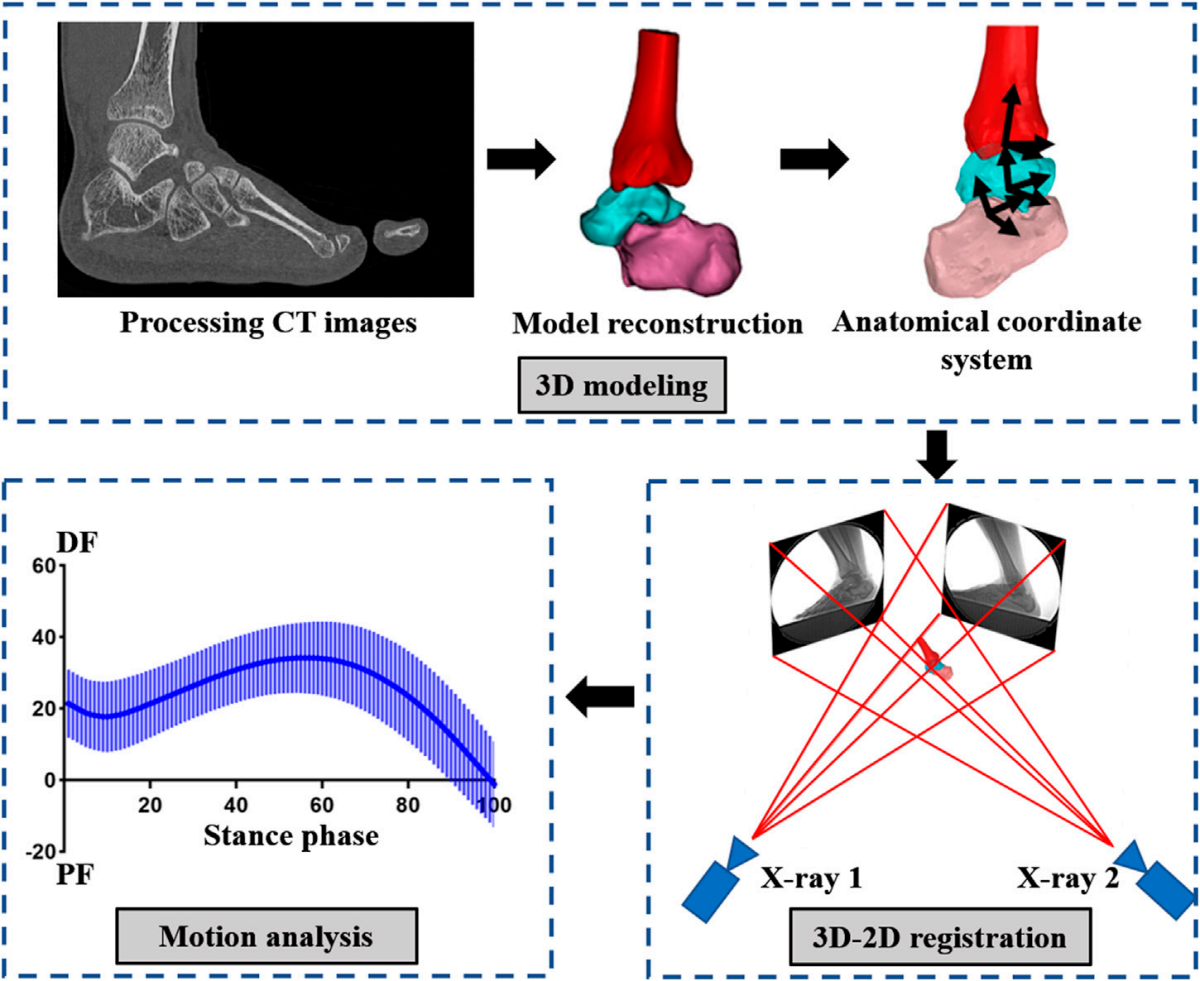
Flowchart of data collection and analysis using DFIS.

DFIS is derived from fluoroscopic imaging technology, which is widely used in the medical field for its penetration and noninvasive characteristics. However, fluoroscopic imaging mainly captures static bone images and thus cannot easily quantify the dynamic movement of humans. Therefore, researchers developed DFIS for the capture of bones and joints *in vivo* by combining fluoroscopic imaging, medical imaging and 3D-2D model registration technologies. This instrument consists of an X-ray fluoroscopic and a data analysis system. The X-ray fluoroscopic imaging system comprises two radiographic source and detector pairs, two mobile manipulators with fluorescence receivers and intensifiers and two matching digital cameras (Figure 2). By relying on computed tomography (CT), X-ray imaging and magnetic resonance imaging (MRI), the motion fluoroscopic image system can accurately quantify the movements of *in vivo* bony structures. The data analysis system is composed of 3D modelling, 3D–2D model registration and a motion analysis system (Figure 3). It is mainly responsible for registering and reconstructing 2D images and 3D models in 3D space to provide the exact position of each bone or joint. It is the basis for the final quantification of joint relative displacement, relative angle, cartilage contact area and cartilage contact stress/strain.

### Technical Preference

Numerous methods have been utilised to quantify human movement (Table 1). Some common methods include motion capture systems, which are extensively used to quantify joint kinematic by tracking reflective markers that have been adhered to the skin at bony landmarks (Roach et al., 2017). However, the measuring accuracy of this method is limited by artificial factors, such as marker location, and nonartificial factors, such as skin and soft tissue artefacts (Bauman and Chang, 2010). Furthermore, this system cannot analyse the movement of a single bone nor obtain the 6DOF motion of joints. Although computer simulation methods, for example, the finite element model (Gültekin et al., 2021), can simulate the 6DOF movement of an independent joint with computer programming, it cannot measure *in vivo* joint kinematics, let alone obtain the real movement of the joints in functional activities, such as walking, jumping and running. Human cadaver studies involving the implantation of steel beads or pressure sensors into the human body can also quantify the movement in a single bone but are invasive and raise ethical concerns. Traditional radiological technologies, such as X-ray plain film, MRI and CT, are also used to track bone positions (Kroupa et al., 2020). However, the subjects in the studies based on these technologies are usually in the supine position, which precludes the evaluation of human movement during activities. DFIS can compensate for the shortcomings of other biomechanical methods by noninvasively capturing the motion of multiple joints independently of other joints in dynamic human activities. DFIS is a new approach that is safe and reliable. Cross et al. (2017) estimated that the effective dose for a foot and ankle CT, plus one biplane fluoroscopic static trial and 10 dynamic trials in the current system, is180 μSv, which is far below the annual occupational limit of the whole-body effective dose of 500,000 μSv established by the US Nuclear Regulatory Commission (Brenner et al., 2003). In term of precision, DFIS demonstrated a bias range of −0.16–0.13 mm and −0.05–0.13°, a precision range of 0.05–0.86 mm and 0.06–0.69° and an overall dynamic root-mean-squared average error of 0.59 mm and 0.71° in static and dynamic trials (Cross et al., 2017).

**TABLE 1:** Comparison of DFIS with other biomechanical methods.

Classification	Methods	*In vivo*	Noninvasive	Six degrees of freedom	Single bone movement	Functional activity
Motion capture system	Reflective markers	Yes	Yes	No	No	Yes
Computer simulation	Finite element model	No	Yes	Yes	Yes	No
	Kinetic model	No	Yes	No	No	No
Human cadaver study	Additional load	No	No	Yes	Yes	No
Implantation	Steel beads	Yes	No	Yes	Yes	Yes
	Pressure sensor	Yes	No	No	No	Yes
	Prostheses with sensors	Yes	No	Yes	No	Yes
Radiological technology	X-ray plain film	Yes	Yes	No	Yes	No
	MRI	Yes	Yes	Yes	Yes	No
	CT	Yes	Yes	Yes	Yes	No
DFIS	3D-2D registration	Yes	Yes	Yes	Yes	Yes

## Dual Fluoroscopic Imaging System Studies on Healthy Populations

### Barefoot Conditions

The quantitative analysis of the kinematics of the foot and ankle joints provides a comprehensive understanding of the weight-bearing mechanisms and laws of ankle movement, ankle function and potential injury mechanisms. In the absence of a suitable location for placing a skin marker around the talus, infrared motion capture systems cannot measure the independent motion of the tibiotalar joint from the subtalar joint (Roach et al., 2016). Instantaneous helical axes (IHAs) are commonly used to investigate the maximum range of motion (RoM) of the tibiotalar joint and subtalar joint in three rotational directions (Akinnola et al., 2020). Arndt et al. (2004) found that during walking, the tibiotalar and subtalar joints have a RoM of inversion/eversion (IN/EV) of 6.3 and 8.3°, respectively, and the dorsiflexion/plantarflexion (DF/PF) of 18.7 and 3.7°, respectively. However, a study using DFIS found that the tibiotalar and subtalar joints have the RoM of 3.8 and 11.3° for IN/EV, respectively, and 16.3 and 8.6° for DF/PF, respectively; these results are different from those obtained from IHAs (Roach et al., 2016). These differences are related to the inability of IHAs to determine the precise position of the bone. Meanwhile, reliable kinematics data for the different stages of dynamic activity are insufficient when the results for IHAs are only for the joint angle at a certain time.

DFIS can noninvasively measure the movement characteristics of a single bone. It has been used to quantify foot and ankle kinematics in healthy populations under barefoot conditions (Table 2). Phan et al. (2019) used DFIS to observe the movement of the transverse tarsal joint during barefoot walking and found that this joint is positioned from the maximum pronation in the early stance phase to the maximum supination in the late stance phase. This action is combined with muscle activities to effectively transmit the push-off force in the late stance phase. This finding provides a new perspective for understanding ankle movement during walking. Additionally, the translation and rotation of the tibiotalar and subtalar joints vary at different stages of the support phase. During the initial landing, the tibiotalar and subtalar joints perform remarkable 6DOF movements, whereas only the subtalar joint performs a remarkable IN/EV and internal/external rotation (IR/ER) at the late support phase (Phan et al., 2018).

**TABLE 2:** Summary of DFIS studies on healthy populations.

Reference	Sample size	Participant (numbers, sex, age)	Anatomical structure	Activity	Biomechanical characteristics
de Asla et al. (2006)	5	Healthy subjects, 4M/1F, 32–43	The subtalar joint and the tibiotalar joint	Non-weight-bearing activity: four complete RoM position; weight-bearing activity: heel strike, midstance, toe-off	Compared with the subtalar joint, the DF/PF in the tibiotalar joint ↑ and the IN/EV ↓
Wan et al. (2008)	6	Healthy subjects, 4M/2F, 24–42	The cartilage of the ankle joint	Non-weight-bearing to fully weight-bearing of ankle joint	During weight-bearing, the cartilage contact strain ↑
Li et al. (2008)	4	Healthy subjects, M, 32–42	The cartilage of the ankle joint	Non-weight bearing to fully weight-bearing of ankle joint	During weight-bearing, the cartilage contact deformation and contact strain during ↑; the increase rate of contact deformation and contact strain ↓
De Asla et al. (2009)	4	Healthy subjects, M, 32–45	ATFL	Non-weight bearing activity: four complete RoM position	From the neutral position to maximal PF of the ankle joint, the length of ATFL ↑; from the neutral position to maximal DF, ATFL ↓; from the maximal pronation to maximal supination, AFTL ↑
Koo et al. (2015)	10	Healthy subjects, M, 21.5 ± 1.9	The subtalar joint and the tibiotalar joint	Walking (1.0 ± 0.1 m/s)	During the stance phase of walking, DF occurs earlier in the subtalar joint than in the tibiotalar joint
Roach et al. (2016)	10	Healthy subjects, 5M/5F, 31.0 ± 7.2	The subtalar joint and the tibiotalar joint	Single-leg, balanced heel rise; walking (0.5 m/s, 1.0 m/s)	During balanced heel rise and walking at 0.5 and 1.0 m/s, DF/PF ↑, IN/EV and IR/ER ↓ in the tibiotalar joint; during walking at 0.5 m/s, the anterior/posterior translation of subtalar joint ↑
Phan et al. (2018)	18	Healthy subjects, M, 23.2 ± 1.8	The subtalar joint and the tibiotalar joint	Walking (self-selected speed)	During the 0–20% of the stance phase, the mean relative speed of tibiotalar joint ↑, IR/ER ↓; the speed of the subtalar joint in 0–10% and 80–90% stance phase ↑ than the rest of the stance phase
Phan et al. (2019)	18	Healthy subjects, M, 23.2 ± 1.8	The midtarsal joint	Walking (self-selected speed)	Before the mid-stance of the walking phase, the midtarsal joint moved towards extreme pronation and performed extreme supination later
Peltz et al. (2014)	12	Amateur runners, 6M/6F, 24.2 ± 4.4	The subtalar joint and the tibiotalar joint	Running (self-selected speed)	During barefoot running, the DF/PF and IR/ER in tibiotalar joint ↑
Hoffman et al. (2015)	12	Amateur runners, 6M/6F, 24.2 ± 4.4	Navicular bone	Running (self-selected speed)	During running with motion control shoes, the navicular drop rate ↓
Campbell et al. (2016)	6	Healthy subjects, M, 37.8 ± 8.6	The ankle joint	Walking (90 steps/min)	During barefoot walking, the PF of ankle joint ↑; the DF/PF and EV occurred later in the stance phase

M = male, F = female, RoM = range of motion, IN/EV = inversion/eversion, DF/PF = dorsiflexion/plantarflexion, IR/ER = internal/external rotation, ATFL = anterior talofibular ligament. “↑” represents that the RoM or the velocity of joint movement is larger or higher. “↓” represents that the RoM or the speed of joint movement is smaller or lower.

Previous DFIS studies have demonstrated that the tibiotalar joint primarily performs DF/PF movements and the subtalar joint primarily performs IN/EV and IR/ER movements in the support phase of walking (de Asla et al., 2006). Roach et al. (2016) discovered that in addition to DF/PF movements, the tibiotalar joint also undergoes slight IN/EV and IR/ER movements, and the subtalar joint performs DF/PF movements in walking and single-leg balanced heel-rise tasks; the PF of the subtalar joint even appears earlier than that of the tibiotalar joint (Koo et al., 2015). Yamaguchi et al. (2009) used a single-plane fluoroscopic imaging system and found that the tibiotalar and subtalar joints execute DF/PF and IN/EV in single-leg heel-rise tasks. The difference in kinematic results indicates that DFIS effectively compensates for the lack of a single-plane perspective for the observation of the 3D motion of the joint and comprehensively analyses a single bone in a dynamic/static status. Furthermore, the tracking data can effectively supplement the previous kinematics results, thereby avoiding the erroneous judgment of movement law as much as possible.

### Shod Conditions

Shod conditions seriously affect the kinematics of the foot and ankle joints (Roberts et al., 2011). For a long time, researchers used various technical means, amongst which motion capture systems were widely used, to identify the relationship between shoes and foot and ankle biomechanics. However, given that the kinematics calculated *via* skin marker motion analysis are visualised by using a generically scaled model, the relationship between individual anatomical features and the ensuing joint motion cannot be discerned (Roach et al., 2016). Meanwhile, in this method, bone movement is mostly calculated by pasting reflective markers on the upper shoe or destroying the shoe structure to paste the markers (Cigoja et al., 2020; Yang et al., 2020). This approach complicates the accurate analysis of the influence of shod conditions on the foot and ankle caused by the unavoidable technical defects of the motion capture system. By contrast, DFIS, which is based on fluoroscopic imaging technology, can directly observe the actual movement of the bone. It thus compensates for the inability of motion capture systems to quantify the bone inside of the shoe and is now the only method that can accurately capture the *in vivo* kinematics of the foot and ankle, even the metatarsophalangeal joint, under shod conditions.

Studies used DFIS to observe the real movement of bones inside shoes and found that shoes limit the PF movement of the ankle joint during walking (Campbell et al., 2016). The remarkably earlier appearance of the DF/PF and EV of the ankle joint under shod conditions than under barefoot conditions indicates that shoes may affect the initiation of foot movements and muscle activation. Furthermore, the RoM of the DF/PF and IR/ER of the tibiotalar joint under barefoot conditions is greater than that when wearing minimalist or motion control shoes during running (Peltz et al., 2014). This finding is inconsistent with a previous result that showed that minimalist shoes have little influence on foot and ankle movements and can simulate barefoot running (Nordin and Dufek, 2020). This inconsistency can be attributed to differences in conditions, such as the type of minimalist shoes used, and may also be related to the inability of previous studies to observe the real movement of the inner bone under shod conditions. Shod conditions also influence the movement of the rest of the foot and ankle joints. For example, a study that utilised DFIS discovered that the navicular drop rate is lower when running in motion control shoes than when wearing minimalist shoes or under barefoot conditions (Hoffman et al., 2015). Thus, wearing motion control shoes is more likely to prevent foot and ankle injuries, such as iliotibial band syndrome, periostitis, bursitis and stress fractures (Benca et al., 2020).

## Dual Fluoroscopic Imaging System Studies on Pathological Populations

The ankle is one of the vulnerable parts of the human body and is always the focus of biomechanics and medicine to prevent ankle injury and accelerate recovery (Tenforde et al., 2016). DFIS can reveal the complex and fine movements in the foot and ankle and has been leveraged to explore the injury and kinematics of these organs in pathological populations (Table 3).

**TABLE 3:** Summary of DFIS studies on pathological populations.

Reference	Sample size	Participant (numbers, sex, age)	Anatomical structure	Activity	Biomechanical characteristics
Caputo et al. (2009)	9	ATFL injury group, 5M/4F, 19–57	The cartilage of the ankle joint	Walking (self-selected speed)	ATFL injury group: IR, anterior and superior translation of talus ↑
Bischof et al. (2010)	7	ATFL injury group, 3M/4F, 33–57	The cartilage of the ankle joint	Stepping onto a force plate	ATFL injury group: cartilage strain↑; the anterior translation and medial translation of the location of peak strain on the injured ankle ↑
Roach et al. (2017)	14	CAI group, 3M/1F, 30.8 ± 4.1; control group, 5M/5F, 30.9 ± 7.2	The subtalar joint and the tibiotalar joint	Single-leg, balanced heel rise; walking (0.5 m/s, 1.0 m/s)	CAI group: IR/ER, IN/EV of the tibiotalar joint and IR/ER of the subtalar joint in balanced heel rise ↓; DF/PF of tibiotalar joint and IR/ER of the subtalar joint during walking at 0.5 m/s ↓; DF/PF, IR/ER, IN/EV of the subtalar joint during walking at 1.0 m/s ↓
Cao et al. (2019a)	30	CAI group, 5M/5F, 24.4 ± 5.4; LAS group, 5M/5F, 25.5 ± 4.6; control group, 6M/4F, 26.4 ± 2.5	The subtalar joint and the tibiotalar joint	Walking (1.0 m/s)	CAI and LAS groups: The anterior/posterior translation of tibiotalar joint during walking ↑; CAI group: The lateral/medial translation and IR/ER of subtalar joints ↑
Cao et al. (2019b)	30	CAI group, 5M/5F, 24.4 ± 5.4; LAS group, 5M/5F, 25.5 ± 4.6; control group, 6M/4F, 26.4 ± 2.5	The subtalar joint and the tibiotalar joint	Stair descent (60 steps/min)	CAI and LAS groups: The IN of tibiotalar joint and subtalar joints ↑; CAI group: The anterior translation of subtalar joints during stair descent ↑
Zhang et al. (2019)	18	CAI group, 3M/5F, 22.4 ± 1.6; control group, 6M/4F, 25.2 ± 1.8	The subtalar joint and the tibiotalar joint	Walking (1.2 m/s)	CAI group: IN/EV of tibiotalar joint and subtalar joint ↓ after wearing an ankle brace
Cao et al. (2019c)	11	CAI group, 6M/5F, 19–39	The subtalar joint and the tibiotalar joint	Walking on a 15° inversion platform (self-selected speed)	CAI group: The IN of tibiotalar joint and anterior translation and PF and IN of subtalar joints after wearing an ankle brace ↓
Balsdon et al. (2016)	18	Six subjects for each normally arched, pes cavus and functional flat foot group, 18–64	The medial longitudinal arch	Walking (self-selected speed); single-limb weight-bearing stance	Functional flat foot group: The angle of MLA ↑; the angle during dynamic activities in all three groups compared with static standing ↑
Balsdon et al. (2019)	18	Six subjects for each normally arched, pes cavus and functional flat foot group, 18–64	The medial longitudinal arch	Walking (self-selected speed)	During walking, the angle of MLA wearing hard CFO and soft CFO ↓

CAI = chronic ankle instability, LAS = lateral ankle sprain, CFO = custom foot orthosis, MLA = medial longitudinal arch. “↑” represents that the RoM or the angle is larger. “↓” represents that the RoM or the angle is smaller.

### Lateral Ankle Sprains

Lateral ankle sprain (LAS) is one of the most common injuries in sports and recreation; it accounts for up to 23% of all athletic injuries (Fong et al., 2009). Patients with LAS have a high probability of developing joint degeneration and chronic symptoms (Bell et al., 2006). Studies based on DFIS have found that the increasing rates of the cartilage contact deformation and contact strains of the ankle joints significantly increase in the early stance phase (Li et al., 2008). This finding indicates that foot injuries, such as LAS, may occur during the early stance phase of walking and proves the hypothesis regarding the mechanism of LAS (Gribble et al., 2016; Delahunt et al., 2018). The anterior talofibular ligament (ATFL), one of the lateral ligaments of the ankle joint, is linked to LAS and osteoarthritis development (Caputo et al., 2009). However, the injury mechanism of ATFL and the relationship between ATFL injury and the kinematics of the foot and ankle joint remains unclear.

A previous study used DFIS to explore the mechanism of ATFL injury and discovered that the ATFL elongates from the neutral position to the maximum PF position and from the maximum ER to the maximum IR (de Asla et al., 2009). This result suggests that ATFL injury may occur during the movement of PF with IR due to the excessive stretching of the ATFL. Caputo et al. (2009) quantified the foot and ankle kinematics of patients with ATFL injury and found that the IN and anterior and superior translation of the talus increase during walking. This increment potentially increases the load of the medial talus cartilage and shear force. Additionally, ATFL injury increases the cartilage deformation of the tibiotalar joint (Bischof et al., 2010). The correspondence between the increase in deformation and the location of osteoarthritis supports the hypothesis that ATFL injury may be potentially associated with osteoarthritis and joint degeneration.

LAS also affects walking kinematics. Compared with healthy controls, patients with LAS exhibit larger tibiotalar anterior/posterior translation during walking and excessive tibiotalar IN during stair descent (Cao et al., 2019a; Cao et al., 2019c). Chronic ankle instability (CAI) is a consequence of the interaction between mechanical and sensorimotor insufficiencies/impairments following acute LAS (Kobayashi and Gamada, 2014; Delahunt et al., 2018). Some researchers used DFIS to analyse the influence of CAI on ankle movement and found that CAI restricts the activities of the tibiotalar and subtalar joints during walking and single-leg balanced heel rise (Roach et al., 2017). This finding indicates that balancing tasks, such as single-leg heel rise, may be one of the best ways to assess the prognosis of patients with CAI because they can reveal additional differences between the foot and ankle kinematics of patients with CAI and the healthy population. The greater IN and anterior translation of the tibiotalar joint during stair descent (Cao et al., 2019a) and greater lateral/medial translation and IN/EV of the subtalar joints in the stance phase of walking shown by patients with CAI relative to those shown by patients with LAS and healthy subjects indicate that lateral ankle injury persists in patients with CAI (Cao et al., 2019c). Meanwhile, previous studies have discovered that patients with CAI experience excessive IN movement in the ankle and that the subtalar joint is mainly responsible for IN/EV in the global movement of the ankle joint (de Asla et al., 2006). Thus, the functional training of the subtalar joint may have considerable clinical importance in the treatment of CAI.

DFIS studies have also found that ankle braces can limit the IN/EV of the tibiotalar and subtalar joints during the stance phase of walking (Zhang et al., 2019). However, ankle braces neither limit anterior translation and the PF of the subtalar joints nor help the RoM of the subtalar joints return to a normal or near-normal level in patients with LAS and CAI (Cao et al., 2019b). This result differs from the findings of previous studies that utilised the traditional motion capture system and showed that ankle braces could limit excessive ankle movement and reduce re-sprain risk for patients with CAI (Dewar et al., 2019). This difference occurred because the traditional motion capture system can only analyse the overall motion of the ankle joint but not the individual motion characteristics of the tibiotalar and subtalar joints. Additional well-designed studies, especially studies using DFIS, are needed to verify the real effect of ankle braces in pathological populations.

### Functional Flat Foot

Functional flat foot is a common orthopaedic problem that may result in disability; its prevalence can reach 20% amongst athletes (Michelson et al., 2002). Compared with people without functional flatfoot deformities, those with functional flatfoot deformities are more likely to develop tendinopathy of the tibialis posterior or Achilles, plantar fasciitis, patellofemoral pain syndrome or even lower back pain as a result of the change in foot structure (Lakstein et al., 2010; Beeson, 2014). Many techniques have been used to assess functional flat foot. The footprint method is one of the most popular and widely used techniques (Wozniacka et al., 2013). However, this method cannot directly measure the real arch movement of people with functional flat feet.

For the past few years, DFIS has been applied to quantify the medial longitudinal arch (MLA) angle and shown that people with functional flat foot have a large MLA angle, and this angle increased during dynamic activities (Balsdon et al., 2016). This result is predictable because the arch of the functional flat foot collapses and the height of the navicular bone is relatively low. In dynamic tasks, the skeletal and ligamentous structures that constitute the MLA play a major role in transferring and dampening forces through their deformation to protect the foot (Caravaggi et al., 2019). Foot orthotics can reduce the angle of MLA in people with functional flat foot and increase the arch height to adjust abnormal foot morphology to a certain extent (Balsdon et al., 2019).

Previous studies have typically used motion capture systems to assess functional flat foot (Su et al., 2017). For example, Jung et al. (2011) found that the MLA angle in healthy people when standing is 145.1 ± 5.5°, which is considerably higher than that measured by DFIS under the same condition (129.2 ± 7.6°) (Balsdon et al., 2016). This difference could be attributed to the subtler motion of the foot arch than that of the rest of the lower extremity kinetic chain. Meanwhile, the size, position and relative displacement to the skin of markers make the arch kinematics measured *via* traditional measurements very different from the real state (Gorton et al., 2009). Therefore, DFIS has a higher potential for clinical application than the traditional measurement method that uses inverse dynamics to identify joint damage mechanisms or disease evaluation by clinicians. Furthermore, DFIS can be exploited to observe *in vivo* bone movement directly for the analysis of potential pathogenic mechanisms and the provision of favourable conditions for the accurate diagnosis and treatment of ankle injury.

Current studies on foot and ankle pathologies have clarified the influence of injuries and abnormal arch on movements from the perspective of kinematics. The role of clinical rehabilitation devices and potential factors that cause arthritis and joint degeneration have also been discussed. However, only a few reports have focused on DFIS combined with kinetic measurements. Therefore, future works should combine kinetic measurements, such as force platform and foot pressure technology, with DFIS to quantify the biomechanical characteristics of foot and ankle movements in a multidimensional manner and understand the relationship between ankle movement and injury.

## Limitations and Future Directions

Although DFIS shows incomparable advantages in evaluating foot and ankle kinematics, its application faces certain limitations and technical difficulties (Table 4). Firstly, the small sample size (10–20 participants) and low-quality study design of previous studies could be main limitations. Meanwhile, the limited shooting scope of DFIS introduces difficulty in investigating the continuous movement of the foot and ankle during a complete gait cycle and therefore complicates the selection of a shooting angle and the test movement of the foot and ankle. Secondly, data analysis in previous works was complex, time-consuming and difficult. Therefore, researchers need to complete the 2D or 3D registration of 3D bone models, which comprise nearly 30 small bones with different shapes, of the foot and ankle with high accuracy and consistency.

**TABLE 4:** Preferences and shortcomings of the DFIS.

Preferences	Shortcomings
1) Noninvasive	1) Ionising radiation
2) High precision and repeatability	2) Limited shooting scope
3) Dynamic capture of a single bone	3) Time-consuming in data analysis
4) Avoiding error from the vibration of skin and soft tissue	—

In future studies, an automatic, high-accuracy registration program for foot and ankle joints must be developed to shorten the data-processing time. Moreover, a specific bone stress algorithm combined with the finite element model should be established to obtain the biomechanical and dynamic load characteristics of *in vivo* bony structures and small joints during foot and ankle movements. Future research can continuously explore the effects of gender, age, running posture, shoe types, ankle orthosis and braces on the foot and ankle kinematics of normal and injured feet. A kinematics database must be created to further identify foot and ankle injuries and diseases, provide rehabilitation plans and potentially serve as a basis for the development of sports equipment and the evaluation of rehabilitation. Given that the traditional kinematic measurements cannot reflect the movement inside segments and joints, DFIS exhibits advantages in observing the subtle and complex movements of bones and joints in the foot and ankle. Future studies could assess joint movement with the prioritisation of DFIS and even verify the kinematics results obtained via traditional measurements.

## Conclusion

Previous studies have shown that DFIS has incomparable advantages in the measurement of joint kinematics over other biomechanical methods. In actual application, DFIS has been used to quantify the 6DOF movements in the tibiotalar joint, subtalar joint and midfoot bone during functional activities. In this review, the influences of shoes and ankle braces were discussed. Meanwhile, the effects of LAS and functional flat foot on joint kinematics were emphasised. This review illustrated that DFIS is a valuable measurement tool that can detect small but substantial differences in the foot and ankle joint kinematics in healthy and pathological populations. In all, this review demonstrated the possibility of using DFIS to expand the knowledge on *in vivo* foot and ankle joints. Future works could further deepen the application of DFIS in biomedical engineering and biomechanics to explore the movements of the foot and ankle joints and even those of the lower extremities in different populations and pathological symptoms.
